# A Case of Bilateral Renal Infarct Due to Left Ventricular Thrombus

**DOI:** 10.7759/cureus.58273

**Published:** 2024-04-14

**Authors:** Varun Daiya, Tushar Sontakke, Sunil Kumar, Sourya Acharya, Khadija F Hamdulay

**Affiliations:** 1 Department of Medicine, Jawaharlal Nehru Medical College, Datta Meghe Institute of Higher Education & Research, Wardha, IND

**Keywords:** thrombo-embolic events, myocardial infarction, lv thrombus, lv clot, anticoagulation

## Abstract

Renal infarction is an uncommon illness that can have serious side effects. Patients may be predisposed to the disease by factors including smoking, atrial fibrillation, thrombus, infective endocarditis, myocardial infarction, and prosthetic valves. Patients are most susceptible from 24 hours to 15 days after myocardial infarction, with an increased rate of left ventricular (LV) thrombus development, which raises the probability of thromboembolic events in the cerebrovascular system and might exacerbate morbidity and mortality rate. This can be diagnosed by two-dimensional echocardiography. Different risk factors can contribute to the development of an LV thrombus. Renal infarcts from LV clots are less common but can occur bilaterally in certain situations. A 30-year-old male diagnosed with anterior wall myocardial infarction presented at our hospital and was suspected to have bilateral renal infarcts, possibly due to the LV thrombi. The patient was managed on anti-thrombolytics and was reported to be doing well at a follow-up of one month.

## Introduction

Renal infarction is very rare, and its diagnosis is delayed or misdiagnosed as the patient may have a vague presentation like renal colic. A prompt diagnosis is necessary, as it may lead to kidney damage [1]. Myocardial infarction, infective endocarditis, thrombus, smoking, atrial fibrillation, and prosthetic valves play a pivotal role as predisposing factors for renal infarction [2]. Left ventricular (LV) thrombus has been commonly reported in patients with ST-elevation myocardial infarction (STEMI), usually after 24 hours. This thrombus may affect systolic function and should be diagnosed earliest with two-dimensional (2D) echocardiography [3]. LV thrombus can be managed, and better outcomes can be achieved with timely intervention. Some risk factors, such as acute coronary syndrome, hypercoagulability, prolonged inflammation, and smoking, can aid in the formation of an LV thrombus. Thromboembolism leads to adverse outcomes, particularly cerebrovascular events [4].

## Case presentation

**Figure 1:**
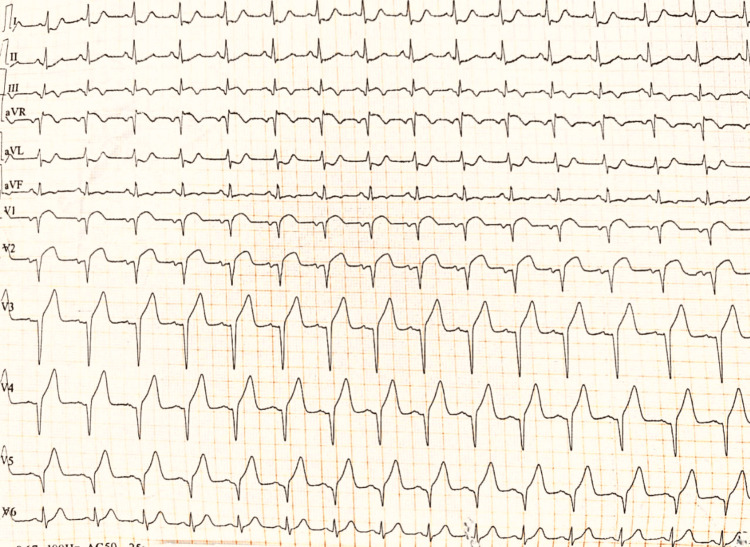
ECG of the patient suggestive of STEMI ST elevation in the lead augmented vector, right V1-V6 STEMI, ST-elevation myocardial infarction

A 30-year-old male presented with chest pain, anxiety, sweating, and palpitations for two days, for which he was admitted to our hospital. There was no associated history of any other major illness, such as hypertension or diabetes. The physical examination was unremarkable. The patient on admission had normal vitals with a respiratory rate of 18/min, a pulse rate of 110 beats/min, and a 97% oxygen saturation level at room air with a blood pressure of 140/90 mmHg. In view of chest pain, an ECG was advised, which was suggestive of an evolved STEMI (Figure 1).

**Figure 2:**
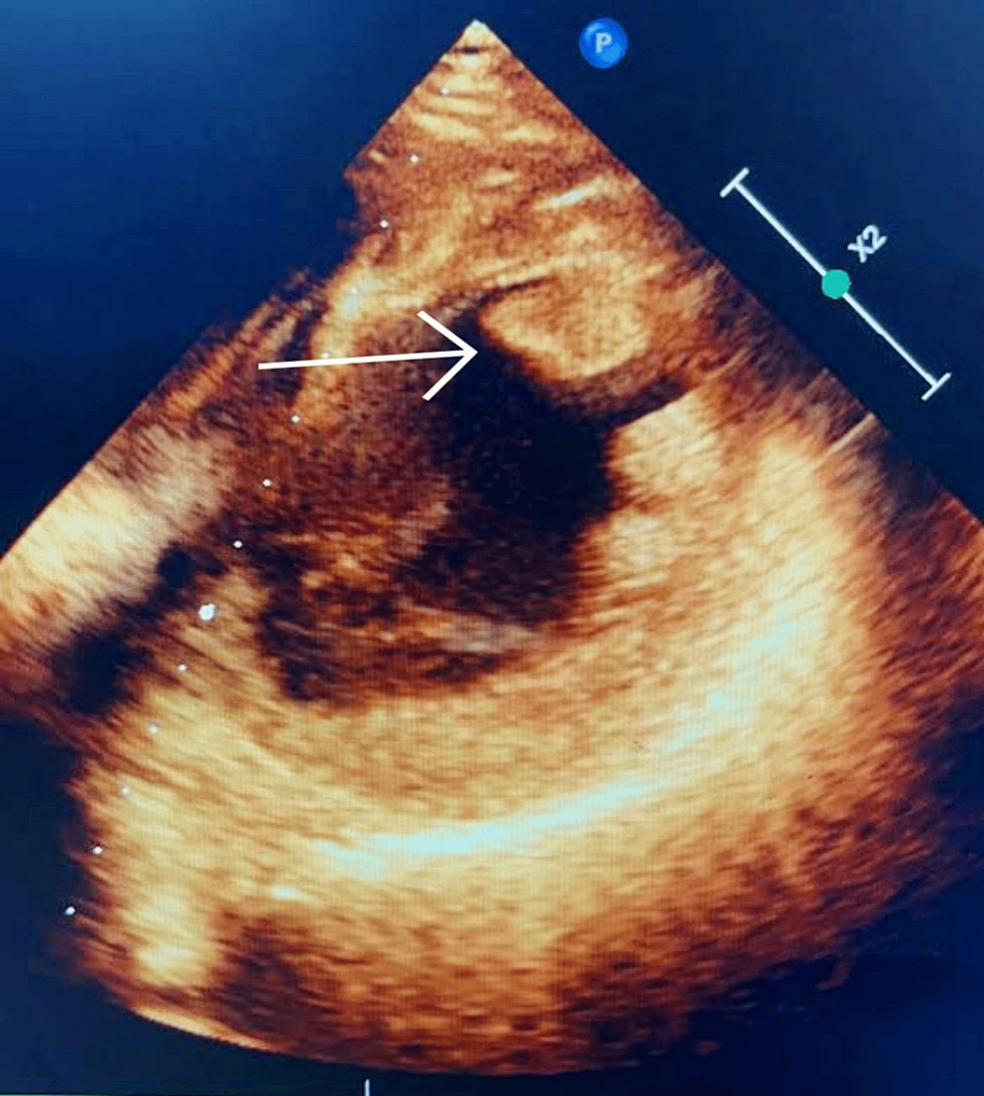
A 2D echo report showing an LV clot The arrow indicates the clot. 2D, two-dimensional; LV, left ventricular

Troponin I was found to be elevated. In view of the anterior wall myocardial infarction, a loading dose of anti-platelets (Ecospirin 300 mg and Clopidogrel 300 mg) was given. A 2D echo report was suggestive of regional wall motion abnormalities, anterior wall hypokinesia, and an LV ejection fraction (LVEF) of 30-40% with an LV clot (Figure 2).

The blood test parameters were within the normal limits, as presented in Table 1.

**Table 1:** Laboratory investigations of the patient

Blood parameter	Value	Reference range
Hemoglobin	13.6 gm%	13-17 gm%
WBC	5,600 cells/cumm	4,000-10,000 cells/cumm
Platelets	2.87 lakh/cumm	1.5-4.1 lakh/cumm
Mean corpuscular volume	78.9 fL	83-101 fL
Alkaline phosphokinase	79 IU/L	44-147 IU/L
Alanine aminotransferases	45 U/L	<50 U/L
Aspartate aminotransferases	47 U/L	17-59 U/L
Albumin	3.5 gm/dL	3.5-5 gm/dL
Total bilirubin	1.0 mg/dL	0.2-1.3 mg/dL
Urea	31 mg/dL	19-43 mg/dL
Creatinine	0.8 mg/dL	0.66-1.25 mg/dL
Sodium	137 mmol/L	137-145 mmol/L
Potassium	4.8 mmol/L	3.5-5.1 mmol/L
CRP	0.2 mg/dL	<0.3 mg/dL
Homocysteine	12 mmol/L	6.6-14.8 mmol/L

**Figure 3:**
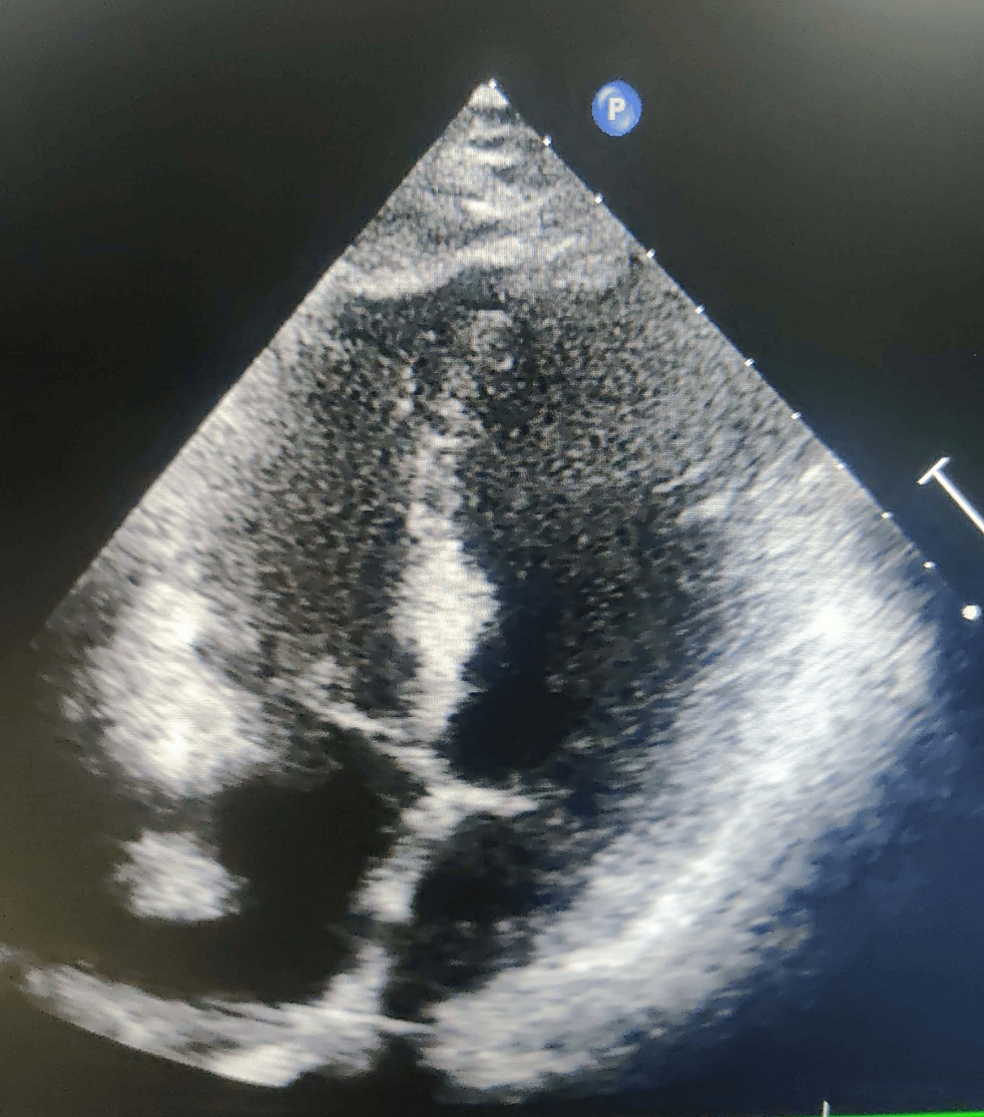
A 2D echo image showing no LV clot 2D, two-dimensional; LV, left ventricular

After 24 hours of admission, the patient developed acute pain in the abdomen with backache. The patient was advised against contrast-enhanced computed tomography (CECT) abdomen and pelvis, which revealed ill-defined hypodense areas in the parenchyma of the upper and middle poles of the right and upper middle and lower poles of the left kidney, featuring the possibility of bilateral renal infarction. A follow-up 2D echo was done, which revealed distal interventricular septum apical lateral wall hypokinesia, mildly dilated left atria and left ventricle, mild mitral regurgitation, no clot or vegetation, LVEF 50%, and moderate LV systolic dysfunction (Figure 3).

**Figure 4:**
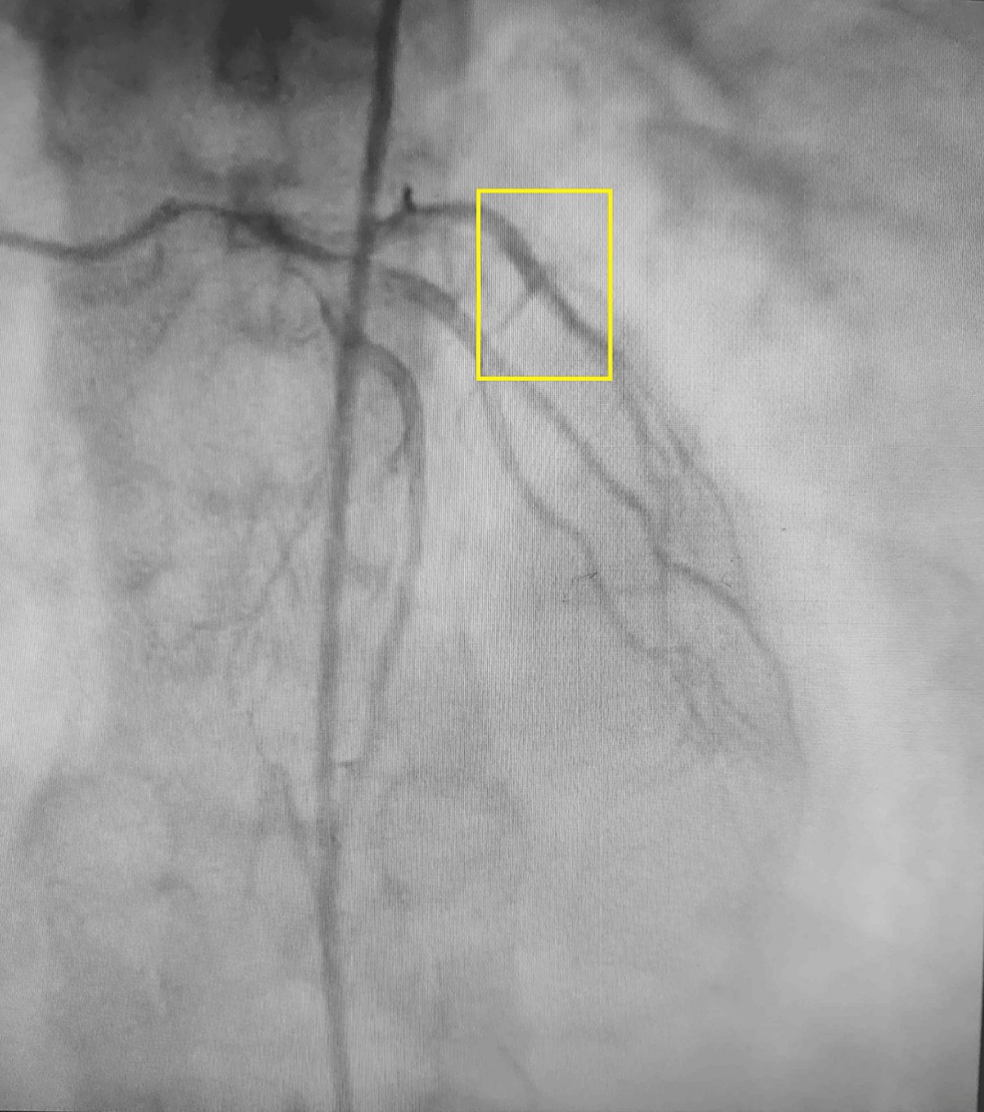
Coronary angiography of the patient presenting a recanalized LAD The highlighted area shows recanalized LAD. LAD, left anterior descending artery

Coronary angiography showed normal epicardial coronaries with a recanalized left anterior descending artery (Figure 4).

**Figure 5:**
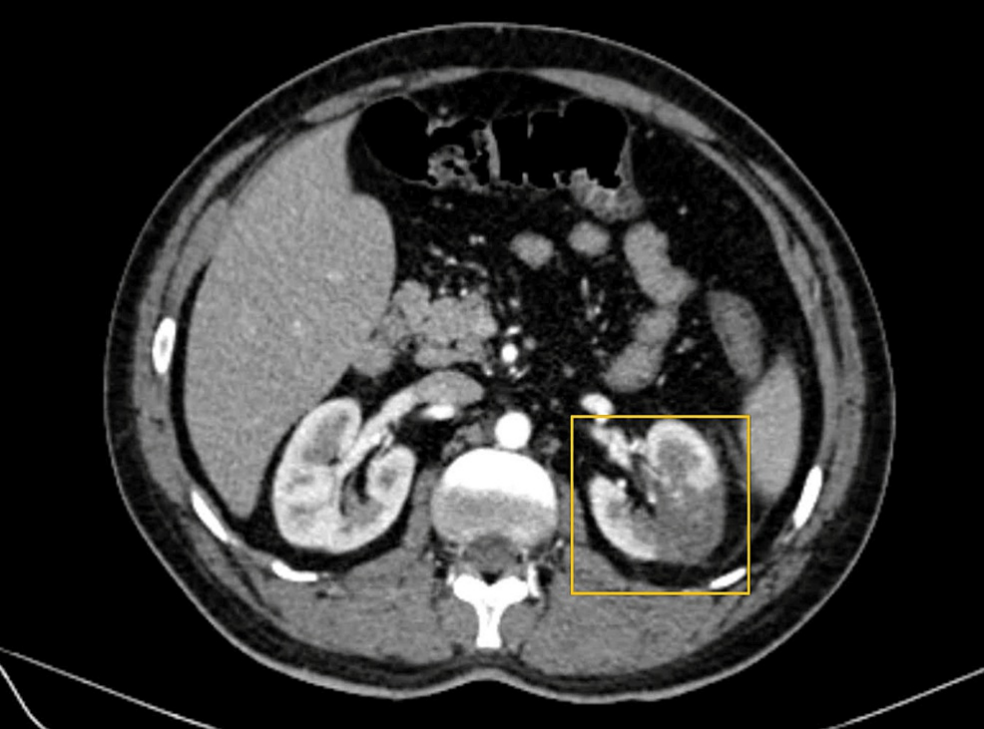
Ill-defined hypodense area in the parenchyma of the left kidney The area highlights the ill-defined hypodense parenchyma of the upper and middle poles of the right kidney.

**Figure 6:**
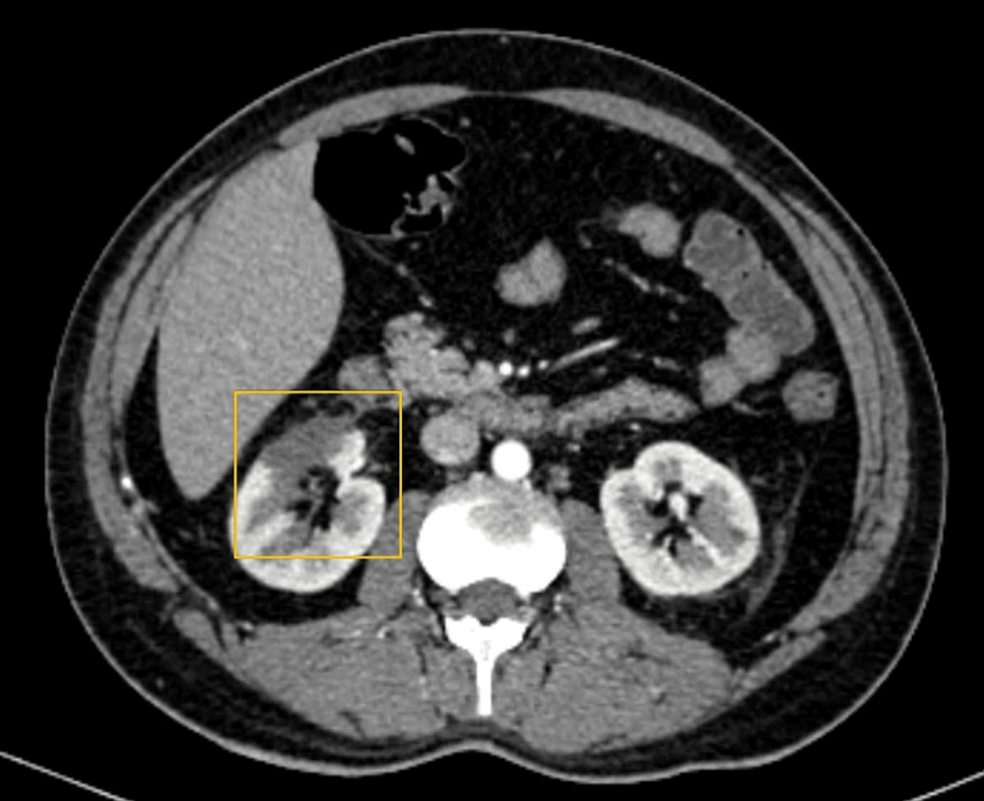
Ill-defined hypodense area in the parenchyma of the right kidney The area highlights ill-defined hypodense parenchyma of the upper, middle, and lower poles of the right kidney, showing no post-contrast enhancement or cortical rim sign.

Based on CECT findings, a final diagnosis of bilateral renal infarction was made following LV thrombus migration (Figure 5, Figure 6).

The patient was started on anti-ischemic drugs, including dual anti-platelet drugs (Ecosprin 75 mg and Clopidogrel 75 mg once a day), Atorvastatin 40 mg once daily, Tab Telmisartan 40 mg once daily, and Enoxaparin 0.4 mg twice a day for four days. The patient became asymptomatic and was discharged after seven days. Follow-up after one month indicated the patient was doing well.

## Discussion

The chances of LV thrombus formation are high in post-myocardial infarction patients for up to 15 days. LV thrombi can increase the chances of thromboembolic events, especially in the cerebrovascular system. Research studies report embolic events post-myocardial infarction ranging approximately from 6% to 86% [5]. In our case, the possible etiology is thromboembolism due to unnoticed atrial fibrillation. Atrial fibrillation predisposes to thromboembolism; however, in post-MI patients, it is poorly contracting LV segments, which leads to stasis of blood and thrombus formation. There are cases reported to have an unnoticed acute episode of atrial fibrillation, which has been noted as an underlying cause of significant morbidities such as embolic events and cerebrovascular stroke [6]. Also, LV clots are less known to cause renal infarcts, which are not observed in 2D echo, leading to a partial diagnosis of clots being resolved by thrombolytic agents.

Despite this rarity, we observed bilateral renal infarcts in this case. Based on CECT findings, a probable conclusion of an LV clot causing bilateral renal infarction was made. The majority of the patients face adverse cerebrovascular events such as a stroke caused by LV thrombus after myocardial infarction, acute coronary syndrome, or open heart surgery as compared to the development of renal infarcts [4], although a few case reports have mentioned patients suffering renal infarction as a result of an embolic event caused by LV thrombus [2]. Usual clinical presentations can be noted as abdominal pain associated with or without vomiting, hematuria, and raised blood pressure, with serum creatinine and urea levels that might or might not be affected [7]. Serpentine thrombus seen in the atria is very pathognomonic of thrombus being dislodged from the deep veins of the lower limbs and can dislodge to other organs [8,9]. A higher rate of morbidity and mortality and unfavorable short-term outcomes are noted in myocardial infarction patients with already-present or developed kidney injuries [10]. The pathogenesis of LV thrombus is usually described by Virchow’s triad: myocardial injury, hypercoagulability, and blood stasis. It is found to be associated with mortality, stroke, renal impairment, and significant adverse cardiovascular events. The risk of developing LV thrombi and related adverse events can be reduced post-myocardial infarction by anticoagulant therapy (vitamin K antagonists and direct oral anticoagulants) for three to six months, with surgical intervention when required [11,12].

## Conclusions

Renal infarction is a rare entity that is often misdiagnosed as abdominal colic; however, this should be suspected in patients with coronary artery disease who present with abdominal pain. Early and prompt diagnosis of LV thrombus can reduce a lot of complications in the form of its dislodgement and propagation to another site like the kidney, thus reducing morbidity. This was the first case of bilateral renal infarction secondary to thromboembolic phenomena in a patient with myocardial infarction.
